# Deep Digital Phenotyping and Digital Twins for Precision Health: Time to Dig Deeper

**DOI:** 10.2196/16770

**Published:** 2020-03-03

**Authors:** Guy Fagherazzi

**Affiliations:** 1 Luxembourg Institute of Health Department of Population Health Digital Epidemiology Hub Strassen Luxembourg

**Keywords:** digital health, digital epidemiology, deep digital phenotyping, digital orthodoxy, precision medicine, precision health, personalized medicine, digital phenotyping, precision prevention, big data, omics, digitosome, data lake, digital cohort

## Abstract

This viewpoint describes the urgent need for more large-scale, deep digital phenotyping to advance toward precision health. It describes why and how to combine real-world digital data with clinical data and omics features to identify someone’s digital twin, and how to finally enter the era of patient-centered care and modify the way we view disease management and prevention.

## Introduction

It has been said that there are “a hundred ways of being diabetic,” but I could argue that there are much more, which is true for virtually any chronic disease. The more we advance in the understanding of a disease, from a biological, clinical, genetic, epidemiological, sociological, behavioral, or psychological point of view, the more we uncover the complexity of medical conditions that health care systems will then have to prevent, treat, and manage.

## Precision Medicine is Much More Than a Matter of Genetic Features

Precision medicine has been defined as [1]:

An emerging approach for disease treatment and prevention that takes into account individual variability in genes, environment, and lifestyle for each person.

Recent achievements have been made in the field of precision oncology [2], but so far, only a small proportion of patients can benefit from personalized treatment each year. Currently, the focus is on the use of genetic or molecular markers to stratify diagnoses and corresponding treatment strategies. However, most are still in the discovery stage in mice models or *in silico*, even if some commercial companies are already starting to use them [3].

How can precision medicine claim to involve patients in their care [4] if it ignores the data they generate in real life? A personalized therapeutic strategy could theoretically suit someone’s biological or genetic phenotype but could fail because of their level of stress, dietary habits, working or living environment, or their cultural background. However, from an economic perspective, we should expect these costly therapeutics (sometimes up to tens of thousands of dollars a month per patient in oncology) to have the best return on investment in terms of compliance and success rate [5].

To achieve the ultimate goal of precision health, which is to match one individual, given their unique profile, with their one, best, medical, therapeutic, and preventive strategy, I argue that we will have to invest in the concept of deep digital phenotyping.

## Deep Digital Phenotyping Is the Missing Link

**Figure 1:**
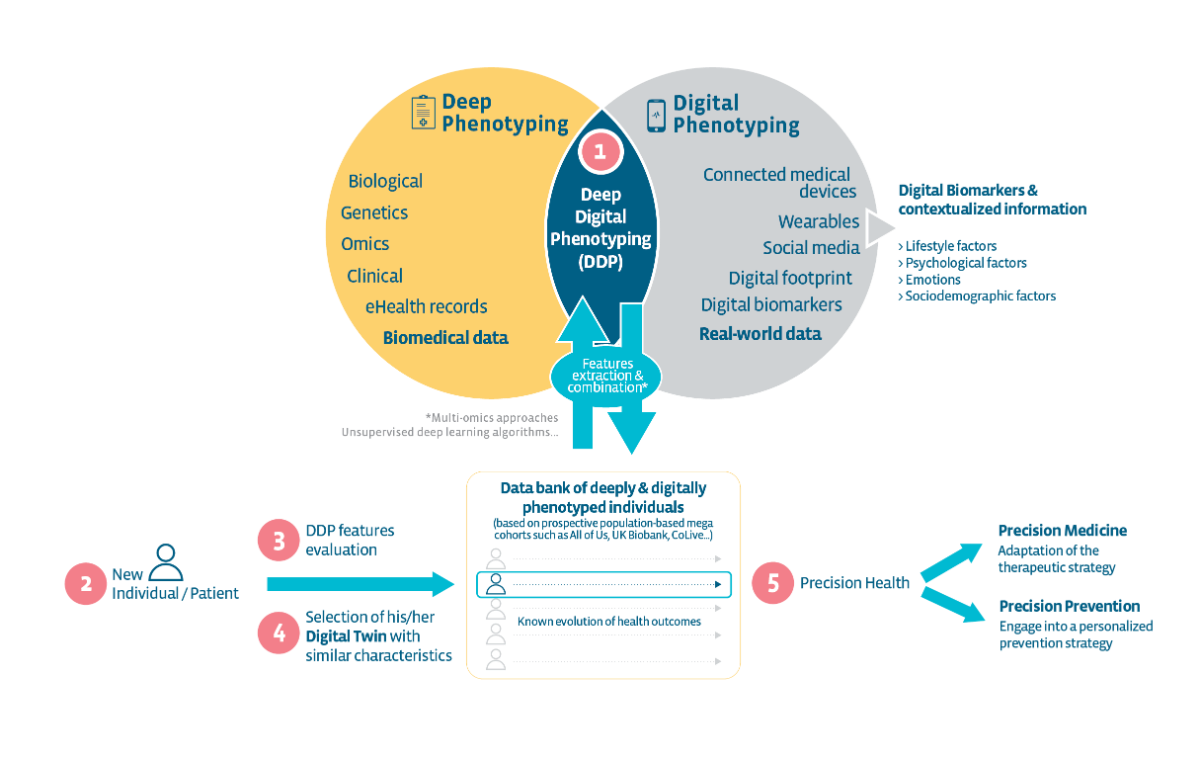
The concept of deep digital phenotyping and digital twin identification for precision health.

Achieving the true purpose of precision health requires integrating, from scratch, features from the “digitosome” (ie, all data generated digitally by individuals online or by their smartphones or connected devices) [6]. Deep digital phenotyping is the combination of deep phenotyping (defined for almost a decade now as the “precise and comprehensive analysis of phenotypic abnormalities in which the individual components of the phenotype are observed and described”) [7], with digital phenotyping, (defined as the moment-by-moment quantification of the individual-level human phenotype *in situ* using data from personal digital devices [8]). If clinically relevant [9], the power of digital data [10] will give us insights, usually in an automated and objective way, about the lifestyle, psychological state, sociodemographics, and environment of a given individual and thus will help capture the bigger picture and reach the full potential of precision health (see Figure 1). Digital phenotyping has already proven relevant in a few areas, such as psychiatry [11] or cardiovascular diseases [12].

Surprisingly, despite the relative ease of acquisition, high volume, and low cost and burden for the individual, these types of data are often neglected and not exploited to characterize the phenotypes of the patient. Do not forget: a person living with diabetes spends roughly 6 hours per year face-to-face with health care professionals and takes more than 600 hours per year to manage (generating data about) their disease(s) by themselves.

Large clinical and epidemiological initiatives can now gather details about disease manifestations, risk factors, and health determinants in a more individualized and detailed way, and use advanced algorithms to integrate data on human behaviors and human-machine interactions through smartphones or connected devices. For example, tracking digital heart rate variability (HRV) as a marker of stress [13] and optimizing drug intake when HRV is at its peak.

## Let Us Meet Our Digital Twin

We have known for some time that “one size fits all” strategies are not efficient therapeutically or preventively [14]. To observe a true leap forward in the domain of precision health, I encourage developing large data banks of “digital twins.” The digital twin is a very new concept in health research and comes from the industrial world, where a digital replica of a physical entity is virtually recreated, with similar elements and dynamics, to perform real-time optimization and testing. The use of digital twins can be extended to the medical field, the elements being features from deep digital phenotyping and the dynamics being the evolution of health outcomes over time. Thus, a digital twin would be a virtual patient with similar or close characteristics as a new patient seen during a clinical visit, and for whom the health status, risks of complications, and disease evolutions are known. This new patient will have a digital twin represented by the average characteristics of its closest cluster group, obtained thanks to deep digital phenotyping.

## Methodology and Ethical Concerns

It seems this is the right moment to implement this digital twin concept. From multi-omics approaches to unsupervised deep learning algorithms, along with the proper computational power, we now have the appropriate tools to deal with the diversity and quantity of information and move from coarsely stratified groups to refined, small groups of individuals defined by numerous features. Methods such as variational autoencoders, an unsupervised deep learning framework, can be used to learn latent representations to cluster and identify deep digital phenotyping patterns [15], or uniform manifold approximation and projection could also be used, which is a dimensionality reduction technique for machine learning [16]. This is on top of using hierarchical agglomerative cluster analyses or k-means clustering to identify refined subgroups of individuals whose detailed characteristics can be averaged to provide someone’s digital twin.

The main challenge to address here is accessing large populations and their detailed information. This will be resolved in the short term with the development of mega cohorts (such as the All of Us Research Program, the UK Biobank, CoLive Diabetes, etc) and other prospective digital health data lakes and big data infrastructure, which will soon provide both deep digital phenotyping of volunteers enrolled in these initiatives and the evolution of their health outcomes.

Going so deep in the phenotyping of populations will also raise ethical and data security concerns. Appropriate clinical and research practices will have to be updated and extended, in parallel with medical and technological evolution, without preventing innovation and ensuring that it will benefit most people. Privacy by design and by default, pseudonymization, traceability, and data portability, key elements from the General Data Protection Regulation [17] or European guidelines on data security for Big Data projects [18], should be the standard of research and included from the beginning in the study and the information technology infrastructure associated with it. Also, conducting transparent research, obtaining informed consent, including participants at every stage of the research, communicating continuously on the different uses of the data collected, and going back to the community and the lay public will ensure trust in deep digital phenotyping methods to combine many sources of data on a large population. In parallel, open data and open source practices should particularly be encouraged in this field.

## Let Us Dig Deep!

We are moving from an ancient world where people with diabetes were characterized by only a few measurements of fasting glucose levels or glycated hemoglobin to a world where frontiers between subclinical types of diabetes are being redrawn. Soon, we will also be moving to a future where we will be able to deeply phenotype individuals with thousands of points of combined clinical, biological, genetic, sociological, psychological, and real-world digital parameters, which will profoundly change the way we characterize patients, and how we understand and contextualize the various forms of diseases. This is where modern epidemiology, combined with computer science, data science, and behavioral psychology, will play a significant role in medical research.

Ultimately, we will enter the era of true precision health and patient-centered care and modify the way we consider disease management and prevention, through the identification of someone’s digital twins. This will augment the capabilities of health care professionals and empower patients by fine-tuning disease management, treatments, and devices to use, as well as biomarkers to monitor.

## Abbreviations

HRV: heart rate variability

## References

[1] Psaty BM, Dekkers OM, Cooper RS (2018). Comparison of 2 Treatment Models: Precision Medicine and Preventive Medicine. JAMA.

[2] Wise HC, Solit DB (2019). Precision Oncology: Three Small Steps Forward. Cancer Cell.

[3] Muse ED, Topol EJ (2019). Digital orthodoxy of human data collection. The Lancet.

[4] Wynn RM, Adams KT, Kowalski RL, Shivega WG, Ratwani RM, Miller KE (2018). The Patient in Precision Medicine: A Systematic Review Examining Evaluations of Patient-Facing Materials. J Healthc Eng.

[5] Kasztura M, Richard A, Bempong N, Loncar D, Flahault A (2019). Cost-effectiveness of precision medicine: a scoping review. Int J Public Health.

[6] Fagherazzi G, Ravaud P (2019). Digital diabetes: Perspectives for diabetes prevention, management and research. Diabetes Metab.

[7] Robinson PN (2012). Deep phenotyping for precision medicine. Hum Mutat.

[8] Torous John, Kiang Mathew V, Lorme Jeanette, Onnela Jukka-Pekka (2016). New Tools for New Research in Psychiatry: A Scalable and Customizable Platform to Empower Data Driven Smartphone Research. JMIR Ment Health.

[9] Huckvale K, Venkatesh S, Christensen H (2019). Toward clinical digital phenotyping: a timely opportunity to consider purpose, quality, and safety. NPJ Digit Med.

[10] Raballo A (2018). Digital phenotyping: an overarching framework to capture our extended mental states. Lancet Psychiatry.

[11] Lydon-Staley DM, Barnett I, Satterthwaite TD, Bassett DS (2019). Digital phenotyping for psychiatry: Accommodating data and theory with network science methodologies. Curr Opin Biomed Eng.

[12] Teo JX, Davila S, Yang C, Hii AA, Pua CJ, Yap J, Tan SY, Sahlén Anders, Chin CW, Teh BT, Rozen SG, Cook SA, Yeo KK, Tan P, Lim WK (2019). Digital phenotyping by consumer wearables identifies sleep-associated markers of cardiovascular disease risk and biological aging. Commun Biol.

[13] Kim H, Cheon E, Bai D, Lee YH, Koo B (2018). Stress and Heart Rate Variability: A Meta-Analysis and Review of the Literature. Psychiatry Investig.

[14] Hardeman W, Houghton J, Lane K, Jones A, Naughton F (2019). A systematic review of just-in-time adaptive interventions (JITAIs) to promote physical activity. Int J Behav Nutr Phys Act.

[15] Wang Zhenxing, Wang Yadong (2019). Extracting a biologically latent space of lung cancer epigenetics with variational autoencoders. BMC Bioinformatics.

[16] Sánchez-Rico Marina, Alvarado Jesús M (2019). A Machine Learning Approach for Studying the Comorbidities of Complex Diagnoses. Behav Sci (Basel).

[17] Chico V (2018). The impact of the General Data Protection Regulation on health research. Br Med Bull.

[18] Ienca M, Ferretti A, Hurst S, Puhan M, Lovis C, Vayena E (2018). Considerations for ethics review of big data health research: A scoping review. PLoS One.

